# Impact of a Papillomavirus Vaccination Promotion Program in Middle School: Study Protocol for a Cluster Controlled Trial

**DOI:** 10.2196/35695

**Published:** 2022-06-13

**Authors:** Phuong Lien Tran, Emmanuel Chirpaz, Malik Boukerrou, Antoine Bertolotti

**Affiliations:** 1 Department of Gynecology and Obstetrics University Hospital of St Pierre Saint Pierre, Réunion France; 2 Centre d’Etudes Périnatales de l’Océan Indien University Hospital of St Pierre St Pierre France; 3 Registre des Cancers Centre Hospitalier Universitaire de La Réunion St Denis, Réunion France; 4 Department of Dermatology and Infectious Diseases University Hospital of St Pierre St Pierre, Réunion France

**Keywords:** HPV vaccine, vaccination program, middle school, school, student, women's health, sexual health, cervical cancer, vaccination, papillomavirus, vaccine, public education, patient education, community education, promotion, program, youth, children, protocol, mortality, uterine cervical cancer, cancer, HPV, health promotion, girls, school, intervention, parent, training

## Abstract

**Background:**

On Reunion Island, incidence and mortality for uterine cervical cancer is high, yet coverage rate for human papillomavirus (HPV) vaccination is low.

**Objective:**

The main objective of the study is to evaluate the impact of a health promotion program promoting HPV vaccination on the proportion of middle school girls who complete the full HPV vaccination schedule (2 or 3 doses) by the end of school year.

**Methods:**

This study is a cluster controlled intervention study using a superiority design. A combined health promotion program will be offered containing information to students and parents, training of general practitioners, and free school-based vaccination (in a “health bus”). Children who attend this program will constitute the intervention group and will be compared to children from another middle school who will not attend the program constituting the control group.

**Results:**

Recruitment began in October 2020. In the intervention school, of 780 students, 245 were randomly selected in the 12 classes. In the control school, 259 students out of 834 were randomly selected.

**Conclusions:**

In this study, we explore the impact of a health promotion program combining information toward students, parents, and general practitioners with free school-based vaccination. We expect a significantly higher HPV vaccination coverage in the intervention school as compared to the control school, whether it be among girls or boys. The final implication would be an extension of this program in all middle schools on the Island and thus an increase in HPV vaccination coverage.

**Trial Registration:**

ClinicalTrials.gov NCT04459221; https://clinicaltrials.gov/ct2/show/NCT04459221

**International Registered Report Identifier (IRRID):**

DERR1-10.2196/35695

## Introduction

### Background

On Reunion Island, a French territory located near the eastern coast of Madagascar in the Indian Ocean, uterine cervical cancer is the fourth most common cause of cancer in women, similar to worldwide [1]. However, the standardized incidence rate in 2016 was 8.8 in 100,000 women, 2 times higher than in metropolitan France. The standardized mortality rate follows a similar trend: on Reunion, it accounts for 4.8 in 100,000 women, whereas the metropolitan rate was 1.7 in 100,000 women [2,3].

Cervical cancer results from human papillomavirus (HPV) infection, which is the most common viral sexually transmitted infection. There are more than 100 types of HPV, some of which are high-risk oncogenes, such as HPV 16 and HPV 18, which are responsible for 70% to 80% of invasive cervical cancers [4]. On Reunion Island, most frequent HPV genotypes are HPV 16, HPV 52, HPV 33, and HPV 31, all contained in the nonavalent HPV vaccine [5].

Indeed, prevention of cervical cancer is mainly based on screening by cervical HPV test and on HPV vaccination, which has proven to be effective in reducing the prevalence of HPV transmission but also in reducing the incidence of condyloma and intermediate grade dysplasia [6,7]. Since HPV is mainly transmitted sexually, it is important to vaccinate before the beginning of sexual life.

Because HPV infections can also lead to vulvar, vaginal, penile, anal, or throat cancers, some countries (eg, United States, Canada, Australia, Germany, Austria, Belgium, Italy) recommend gender-neutral vaccination in order to promote herd immunity and reduce circulation of the virus in the general population [6,8]. In France, since December 2019, it is recommended that HPV vaccination should be offered to all children, regardless of their gender, aged 11 to 14 years (2 doses), with catch-up vaccination possible for adolescents aged 15 to 19 years not yet vaccinated (3 doses). Before December 2019, vaccination was only recommended for girls. High levels of vaccination coverage are obtained in countries that vaccinate in schools [9-11].

On Reunion Island, the HPV vaccination coverage rate is the lowest in France, estimated at 8.1% among girls aged 16 years in 2018, while the national average is already low (23.7%) [12].

This low coverage rate on Reunion Island may have several explanations. First, inhabitants seem to be poorly informed about the existence of this vaccine [13]. Moreover, vaccination coverage rates depend on the socioeconomic level of the population. In France, lower rates of HPV vaccination uptake were observed in adolescents with universal health insurance coverage (French equivalent of the US Medicaid program) compared with those not receiving such insurance [14]. Reunion Island is one of the French departments with the highest rates of inhabitants covered by this universal health insurance. Finally, not only is there vaccination hesitancy in general but also most specifically against HPV vaccine, among patients and also among physicians [13]. A total of 41% of Reunion inhabitants hold unfavorable opinions about vaccinations, with the HPV vaccine being among the most frequently cited. Among patients not vaccinated against HPV, 37% stated that the vaccine had not been suggested to them by their doctor, and 7.3% were confronted with doubts expressed by their doctor concerning vaccination in general [13]. However, a systematic review of 79 studies in 15 countries showed that the most important factor influencing HPV vaccination was physician recommendation [15,16]. Indeed, 89.3% of the Reunion population fully trusts their doctor [13]. Therefore, interventions targeting health professionals and especially general practitioners appear to be paramount, especially when combined with interventions targeting the population to be vaccinated [17].

Thus, given the epidemiological situation on Reunion Island (high incidence and mortality for cervical cancer, very low coverage rate for HPV vaccination), we aimed to study the impact of a prevention program against sexually transmitted infections, including pathologies related to HPV, with a program promoting HPV vaccination among young students in middle school.

### Objectives

Hypotheses were as follows: (1) clear and appropriate information for the target population of the vaccination (middle school students aged 9 to 17 years) as well as for their parents will improve their knowledge about HPV vaccination and thus increase their adherence to this vaccination regimen, (2) combining information with vaccination in the school setting will improve coverage, as it will reduce any material obstacles that may prevent the vaccination process, and (3) raising awareness among general practitioners will enable them to better understand the benefits and risks of HPV vaccination and thus encourage families, who naturally trust them, to adhere to the program.

The main objective of the study is to evaluate, in a population of middle school girls on Reunion Island, the impact of a health promotion program on the proportion of middle school girls who complete the full HPV vaccination schedule (2 or 3 doses) by the end of school year.

The program, conducted during school year, will combine: (1) sexual health promotion (students and parents) during classes at school at the beginning of school year, (2) training of general practitioners (who practice in a perimeter of 5 km around the middle school) on HPV vaccination at the beginning of school year, and (3) free school-based vaccination (in a “health bus”) during the academic year.

Secondary objectives in the study population at the end of school year are as follows:

- Assess the impact of the combined health promotion program on the proportion of middle school girls who initiated HPV vaccination (at least 1 dose)
- Assess the acceptability of the HPV vaccination program among middle school boys
- Describe the barriers to HPV vaccination for both girls and boys
- Assess the acceptability of HPV vaccination in the school setting
- Assess the value of setting up a sexual health information point through a health bus
- Evaluate the satisfaction of students, parents, and school workers with the measures put into place
- Evaluate vaccination coverage for different mandatory vaccines according to the current national vaccination calendar

The aim of this study was to evaluate whether a health promotion program combining information and free school-based vaccination could raise HPV vaccination coverage.

## Methods

### Trial Design

This study is a cluster controlled intervention study using a superiority design. Children who will attend the combined health promotion program will constitute the intervention group and will be compared with children who will not attend the program (as is currently the case in all French middle schools), who will constitute the control group.

### Study Setting

This trial will concern Reunion Island in order to investigate the particular epidemiological situation of HPV on the island, even if the results of this study are expected to be applicable to other French regions.

The 2 arms of the trial will be designed to have the most comparable populations and to avoid any risk of contamination between the 2 arms or having general practitioners taking care of children in both schools.

We have thus chosen to carry out a cluster trial. The 2 groups (intervention group and control group) will be selected from a middle schools located in each of 2 cities. In each of the schools, we will randomly draw 3 classes in each grade level (6th, 7th, 8th, and 9th grade) to have a balanced number of students in each arm (see sample size). Thus, 12 classes will be selected for each school.

Provided that there is a relationship between socioeconomic status and vaccination coverage, it was decided to focus the study only on middle schools in priority education zones, which theoretically enroll a population in which HPV vaccination coverage is the lowest. On Reunion Island, 21 middle schools are classified as priority education zones, spread over 7 cities. In agreement with the head of the academy and the school directors, 2 schools have been designated among the abovementioned middle schools: the intervention school will be Paul Hermann Middle School, located in St Pierre, and the control school will be Plateau Goyave Middle School, located in St Louis.

These choices are based on the schools’ ability to participate in this research, their geographical location, and the ability to park the health bus at or in the immediate surroundings of the school of the intervention group. The health bus will be provided by the Association d’Education Thérapeutique et d’Intervention Sociale (ASETIS, or Association for Therapeutic Education and Social Intervention), existing since 1996 and recognized as being of public interest.

### Eligibility Criteria

Inclusion criteria are as follows: enrolled in one of the classes randomly selected in the 2 middle schools designated, affiliated with or benefiting from a social security system, who will agree to participate in the study and whose parents or holders of parental authority will sign a free, informed, and written consent. Exclusion criteria (intervention group only) are as follows: hypersensitivity to the active substances or to one of the excipients of the vaccine (Gardasil 9), a permanent contraindication to vaccination, pregnant or breastfeeding (based on self-reporting), already initiated HPV vaccination (complete or incomplete schedule), or eligible to participate for collection of data but not for vaccination in the health bus; students with an incomplete vaccination schedule will be referred to their general practitioner to complete the missing doses. Vaccinations will be performed by a junior doctor under the supervision of a senior doctor.

### Intervention Description

#### Intervention Group

Meetings with parents in the intervention middle school will be scheduled at the beginning of school year to inform parents about HPV vaccination and explain this study to them. Consent forms will be collected during these meetings. If assemblies are forbidden by the government because of COVID-19, information meetings for parents will be cancelled. Instead, 6 interventions will be planned.

##### August-September: Program Information Sent Home to Parents via Students

Written information adapted to student age about HPV vaccination, and documents outlining objectives, interventions, constraints, foreseeable risks and expected benefits of the research, and the rights of the participants in this research context will be sent with children to give to their parents. Consent form to participate to the study to be signed by both parents or holders of parental authority and a sociodemographical questionnaire with questions about HPV knowledge will also be included.

##### October-November: Contact With Parents and Return of Information to School

Investigation team will call each authority holder individually by telephone to inform them about HPV vaccination, the study, its objective, nature of the constraints, and foreseeable risks and expected benefits of the research. The team will also remind them of the rights of participants in research and will check the eligibility criteria. Finally, when possible, the team will collect their oral consent. Parents will be asked to place the documents (consent form and sociodemographic questionnaire) in an envelope and seal it before returning it to the main teacher for reasons of data confidentiality. Documents will then be collected by the investigation team.

##### November-December: Data Collection and Student Information Sessions About Sexual Health and Vaccination

Children in the selected classes will be asked to bring in their health record on a specific date, along with the above mentioned documents, for those who forgot to return the envelope to the main teacher previously. On that day, an investigator will collect data necessary for the study in the health records (especially vaccination data) for children for whom consent form was signed by the parents. During this time, an information session about sexually transmitted diseases and vaccination will be given in class, lasting approximately 1 hour and adapted to the level of understanding (according to grade and age), in partnership with teachers. Health records will be immediately returned to the students.

##### November-December: General Practitioner Information Dissemination

A total of 88 general practitioners working in a radius of 5 km around Paul Hermann Middle School will be sent an information leaflet about HPV vaccination and cervical cancer prevention, including the latest literature review, and information about this study. If meetings are forbidden, general practitioners will be invited to a video conference call, “Around HPV,” at the beginning of school year.

##### December, February, and May (3 Campaigns): HPV Vaccination in the Health Bus

Free HPV vaccination will be offered in a health bus for girls and boys. The bus will be parked in the playground, inside the school grounds, allowing students to go there during breaks, lunchtime, or after school. Vaccination periods will be predefined, so that the recommended HPV vaccination schedules can be followed.

Vaccinations will be performed by the medical staff of the University Hospital of Reunion Island (a junior doctor under the supervision of a senior doctor) after informed consent to vaccination signed by either parents or holders of parental authority, who are invited to come along into the bus with their child.

Vaccination will be performed with nonavalent HPV vaccine. The proposed schedule is the one recommended by the marketing authorization: children aged 9 to 14 years (girl or boy): 2-dose schedule (intramuscular), with the second dose to be administered between 5 and 13 months after the first dose; children aged 15 years and older (girl or boy): 3-dose schedule (intramuscular), with the second dose to be administered at least 1 month after the first and the third at least 3 months after the second, with all 3 doses to be administered within 1 year. The vaccine label data will be documented in the health record.

Before vaccination, absence of contraindications will be checked. In case of high fever or acute illness, the vaccination will be postponed and offered at a later date. Vaccinated persons will be monitored for at least 15 minutes after vaccination in the presence of medical staff because of adverse effects that may occur in the direct aftermath of the injection (rare anaphylactic reactions, syncope (fainting) sometimes associated with falls) or psychogenic reaction to needle injection (neurological signs such as transient blurred vision, paresthesias, and tonic-clonic movements of the limbs during the recovery phase). During campaigns in February and May, the first dose of vaccination can be offered, although children will be asked to return to their general practitioner for subsequent doses.

The health bus system will be implemented as part of this study. Two students will be able to be vaccinated at a time. A child will never be left alone with an adult inside the bus; there will always be a minimum of 2 adults present. Students can take advantage of this special time on the bus to receive personalized information on sexuality and obtain free condoms.

##### June: Data Collection in Randomly Selected Classes

At the end of school year (June), an investigator will collect data from health records at a specific time during class. In particular, the researcher will look for the presence of de novo HPV vaccination performed by general practitioners outside of the health bus. Signed consent of parents or holders of parental authority will be collected before any intervention in the study (ie, before data collection and before school vaccination is carried out).

Vaccination data, even if not collected at the time of the intervention, can be collected either during the vaccination campaigns, or during the intervention at the end of school year, especially for children whose parents have agreed to participate but not to be vaccinated in the health bus. Indeed, since vaccination dates appear in the health record, it will be possible afterward to know whether pupils were vaccinated before the interventions under study in order to have the vaccination rate at the very beginning of the study.

##### July-September: Evaluation of Satisfaction and Barriers to Vaccination

Research staff will meet with students, parents, members of school staff, and general practitioners who volunteered, and semidirected interviews will be conducted to understand their satisfaction about the study and determine barriers to vaccination.

#### Control Group

In the control middle school, the study will take place at the end of school year (May-June) in 2 stages.

##### Parent Information About the Study

We will organize parent meetings to inform them of the study. If no parental meeting is possible due to the COVID-19 pandemic, parents of children in selected classes will be sent an envelope containing written information about HPV vaccination and information about the study, the sociodemographical questionnaire, and the objection form to participate to the study (data collection of health record). Thus, if the form is returned to a teacher, the investigation team will not be able to access the child’s health record. On the other hand, if no form is returned, it will be considered that parents do not object to data collection.

##### Data Collection and Student Information About Sexual Health and Vaccination

Children in the selected classes will be asked to bring in their health record on a specific date, along with the completed sociodemographic questionnaire and signed informed consent for data collection from parents. On that day, an investigator will collect data necessary for the study (especially vaccination data) in the health records for children for whom no objection form was returned. During this time, an information session about sexually transmitted diseases and vaccination will be given in class, lasting approximately 1 hour and adapted to the level of understanding (according to grade and age), in partnership with teachers. Health records will be immediately returned to the students concerned.

### Participant Timeline

**Figure 1:**
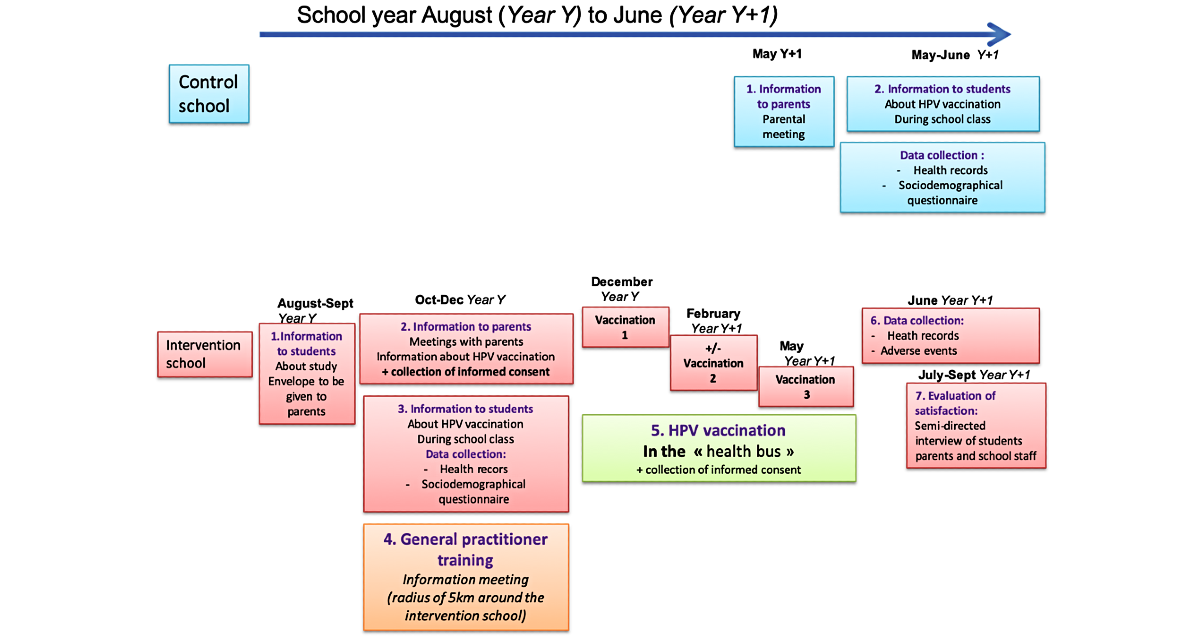
Initial participant timeline. HPV: human papillomavirus.

**Figure 2:**
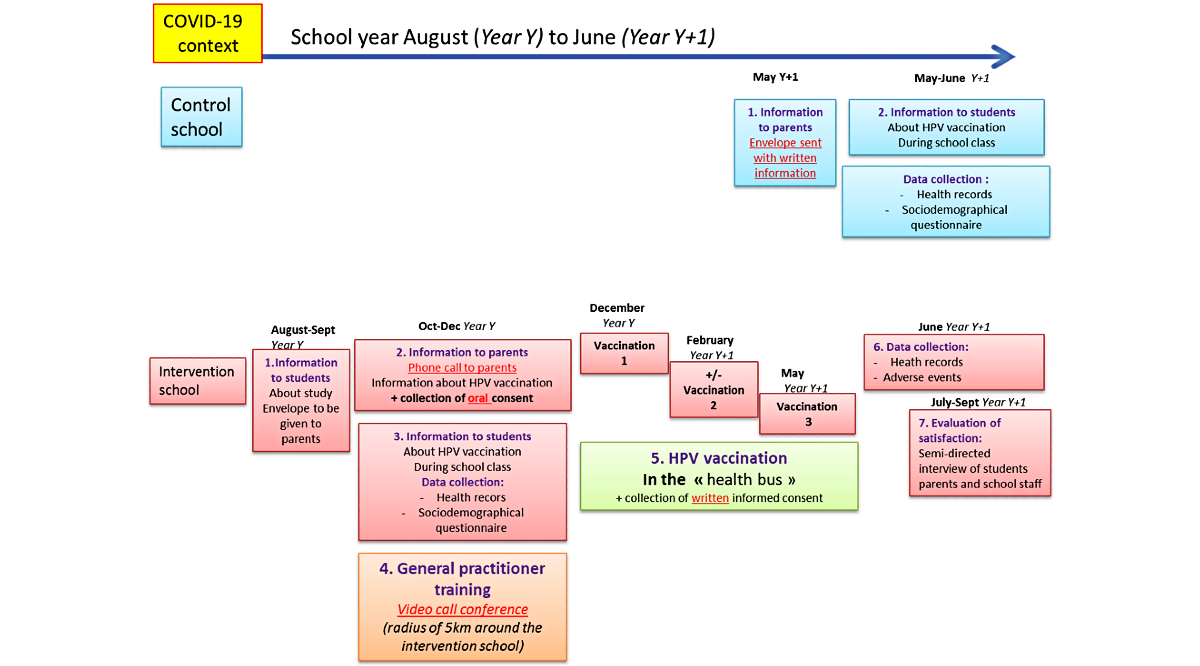
Participant timeline in the context of COVID-19, with meeting restrictions. HPV: human papillomavirus.

Participant timeline is displayed in Figure 1. In case assemblies are forbidden due to COVID-19 pandemic, parental meetings will be cancelled and an alternative participant timeline is displayed in Figure 2.

### Data Collection and Management

Data will be collected in a paper observation book after consent is signed by parents or holders of parental authority and by students. Data will be collected in the form of self-questionnaires (parents and children). Data concerning vaccination status at inclusion and at the end of the study were checked in the health record by the investigation team.

Data will be collected in paper format and will be entered into an electronic case report form (Ennov Clinical) by a clinical study technician. Data will be saved daily. A data validation plan, defined jointly by the principal investigator and the Methodology and Data Management Center (from the University Hospital of Reunion Island), will be developed and the controls to be performed will be described in detail for each variable. Once data entry is completed, the data will be checked for consistency. Inconsistencies will be reported in the CS Test module of Ennov Clinical. The data freeze/unfreeze process will be performed according to the procedure set up in the Methodology and Data Management Center.

### Plans for Storage of Vaccine

University Hospital of Reunion Island will be in charge of purchasing the vaccine doses and providing them, via the central pharmacy, to the ASETIS health bus at the intervention college. There will be no change in product packaging, which was identical to the packaging at the time of purchase: 0.5 mL glass prefilled syringes with needles. Labeling (in accordance with current regulations and good clinical practice) referring to use in clinical research will be affixed to the boxes of vaccines intended for the study. The products will be brought by a staff member of the ASETIS association on the days of school vaccination.

The products will be transported, respecting conditions of conservation of the vaccine (kept between 2 °C and 8 °C and protected from light). Expiration date will be checked before any injection. Accounting and traceability of the doses given will be documented by the doctors administrating the vaccination. The health bus has the capacity to store the vaccine doses in the appropriate conditions in a refrigerator.

The products will be stored at the central pharmacy in University Hospital of Reunion Island and in the health bus on vaccination days. The unused doses during the first school vaccination campaign (first dose) will be returned to the hospital central pharmacy to be used during the following campaigns. At the end of the vaccination campaigns, the unused or expired doses will be destroyed according to the regulations in force.

### Statistical Analyses

#### Sample Size

To our knowledge, there are no recent studies that have evaluated the impact of school-based vaccination on HPV vaccination coverage rates. An experimental catch-up vaccination program (diphtheria, tetanus, poliomyelitis, pertussis, measles, mumps, rubella, meningococcus C, hepatitis B, and HPV) in schools in the Vosges (Eastern France) [18] showed a participation rate ranging from 42.9% in the first year to 29% in the second year.

The calculation of the study size is based on the expected proportion of vaccination among girls, as this is the main objective of this study and we have no data for vaccination among boys at this time. Statistical assumptions were as follow:

- Proportion of schoolgirls who have had a complete HPV vaccination schedule at the end of school year of 6% in the control group for all students (compared to the 8.1% expected at age 16 years [12])
- Proportion of schoolgirls with a complete HPV vaccination schedule at the end of the school year of 20% in the intervention group

Thus, 87 girls in each group would need to be included to demonstrate this 14% difference at *α*=.05 and power (1−β=0.80). Assuming 15% nonanalyzable data (lost to follow-up, nonresponse), a total of 103 female students per group would need to be included. As there are roughly as many girls as boys in each class, we would need to include 206 students per group. In order to have an equal representation of each age group, the sampling will be stratified in the grades: 6th, 7th, 8th, and 9th grade. Thus, 52 students per grade and per group should be included.

If we consider that the classes contain an average of 22 students each, a minimum of 3 classes per grade should be randomly selected in each of the 2 middle schools to ensure the minimum necessary recruitment. Thus, randomly selecting 3 classes per grade in each of the 2 middle schools will make it possible to include approximately 132 female students per group and thus to ensure the minimum necessary recruitment. A total of 528 students are expected to be recruited (264 girls [132 per group] and 264 boys [132 per group]). The calculation of the number of subjects required was performed with PASS (version 15, NCSS) software.

#### Sequence Generation

In priority education schools, there are classes called Sections d’enseignement general et professionnel adapté (SEGPA; adapted general and vocational education sections): these classes, from 6th to 9th grade, are integrated into the middle school. They welcome young people who have significant school difficulties that cannot be resolved by academic assistance and support. There is only a small group of students (16 maximum) in each class in order to individualize each student's progress. SEGPA classes should enable students to access at least a professional qualification.

In the Paul Hermann Middle School, there are 9 classes in each grade, including 2 classes of SEGPA per grade. In the Plateau Goyave Middle School, there are 9 classes in 6th grade and 9th grade and 10 classes in 7th and 8th grade, including 2-3 classes of SEGPA per grade. In each of the selected middle schools, 12 classes will be randomly selected in order to have a balanced number of students in each arm.

In order to have an equal representation of each age group, the sampling will be stratified on the grade (6th, 7th, 8th, and 9th grade), and in order to take into account the specificities of SEGPA classes, we decided to stratify on SEGPA classes as well. As the main point in this comparative trial was similarity of the 2 groups compared, we decided to randomly select 1 SEGPA class per grade and 2 non-SEGPA classes per grade. Thus we will include in this trial 256 students from Paul Hermann Middle School (intervention group) and 255 students from Plateau Goyave Middle School (control group).

#### Statistical Methods for Primary and Secondary Outcomes

The aim of this study is to compare clinical outcomes between classes from a middle school sensitized to HPV vaccination through a combined health promotion program (intervention group) and a middle school without any specific action (control group). The null hypothesis is that there is no difference in the groups. For descriptive analyses, qualitative variables will be described in terms of frequencies and percentages with their 95% confidence intervals; quantitative variables will be expressed in terms of means, standard deviations, and 95% confidence intervals or in terms of medians and IQRs (25th and 75th percentiles).

Comparability of groups at inclusion will be checked: bivariate comparisons of categorical variables will be performed by the chi-square test or Fisher exact test, depending on the conditions of application. Bivariate comparisons of means will be performed by the Student *t* test or Mann-Whitney *U* test, depending on the conditions of application. For the analysis of the primary outcome: the proportion of schoolgirls who will have completed the full HPV vaccination regimen at the end of the school year will be compared between the 2 groups (intervention and control) by the chi-square test or Fisher exact test, according to validity conditions.

Concerning secondary outcomes analysis: the proportion of schoolgirls who initiated HPV vaccination (1 dose) by the end of school year will be compared between the 2 groups (intervention and control) by the chi-square test or Fisher exact test according to validity conditions. The proportion of boys who will have completed the full vaccination schedule at the end of school year will be compared between the 2 groups (intervention and control) by the chi-square test or Fisher exact test, according to validity conditions. The proportion of boys who will have initiated the vaccination scheme at the end of the school year will be compared between the 2 groups (intervention and control) by the chi-square test or Fisher exact test, depending on the conditions of validity.

The analysis of barriers to vaccination will describe the causes of nonvaccination reported for students who did not initiate the vaccination schedule. Analyses will be performed for girls and boys separately. We will also compare sociodemographic data, medical history, and health care utilization data between students who initiated HPV vaccination at the end of the school year and those who did not in the intervention group. Bivariate comparisons of percentages will be performed by the chi-square test or Fisher exact test depending on validity conditions. For continuous variables, comparisons will be made using the Student *t* test or the Mann-Whitney *U* test, depending on the conditions of validity. A multivariate analysis by logistic regression will be carried out in order to take into account confounding phenomena: the variable to be explained will be the fact of having initiated vaccination at the end of the school year, and the explanatory variables entered in the model will be the variables for which the significance threshold in bivariate analysis will be ≤.20.

To be determined in the intervention group: among students who initiated HPV vaccination at the end of school year, the proportion of students who used the health bus to initiate this vaccination. Among students who completed the full vaccination schedule at the end of school year, we will determine the proportion who completed all injections on the health bus.

In the intervention group, the proportion of students who used the health bus for sexual health information will be evaluated. In the intervention group, positive and negative points reported by students, their parents, and school staff about this program will be described. Proportion of students up to date for each type of vaccine (according to current vaccination calendar) at the end of school year, in the entire study population as well as in each of the 2 groups (intervention and control), and comparison of these proportions between the 2 groups by the chi-square test or Fisher exact test will be determined, according to validity conditions.

Analyses comparing control group to intervention group will all be performed on an intention-to-treat basis. All hypotheses will be tested with bilateral tests and *α*=.05 and confidence interval calculated at 95%. Analyses will be performed using SAS (version 9.4, SAS Institute Inc) software.

### Ethical Considerations

The sponsor and investigators agree that this research will be conducted in accordance with the law no. 2012-300 of March 5, 2012, relating to research involving human persons; Good Clinical Practices (version 4 of November 9, 2016, and decision of November 24, 2006); and the Declaration of Helsinki [19]. The research is conducted in accordance with this protocol. Except in emergency situations requiring the implementation of specific therapeutic procedures, the investigators undertake to comply with the protocol in all respects, in particular with regard to the collection of consent and the notification and follow-up of serious adverse events.

This research has received the favorable opinion of the research ethics committee (Comité de Protection des personnes (CPP); ethics committee for the protection of individuals) of Ouest II of Angers (No. 20.05.14.35227; 2020/46) and the authorization of the Agence nationale de la sécurité du médicament (ANSM), the French equivalent of the US Food and Drug Administration. The University Hospital of Reunion Island, promotor of this research, has taken out a civil liability insurance policy with the hospital insurance company Société hospitalière d’assurance mutuelle (no. 158958) in accordance with the provisions of the public health code.

The data recorded during this research are subject to computerized processing at the University Hospital of Reunion Island, responsible for data processing in compliance with the law no. 78-17 of January 6, 1978, relating to data processing, files, and freedoms modified by the law 2004-801 of August 6, 2004 and modified by the law no. 2018-493 of June 20, 2018. This research falls within the framework of the reference methodology (RM-001) in application of the provisions of Article 54 paragraph 5 of the amended Act of January 6, 1978, relating to information, files, and freedoms. This change was approved by decision of January 5, 2006, updated on July 21, 2016. The University Hospital of Reunion Island, responsible for data processing, has signed a commitment to comply with this reference methodology. The research sponsor undertakes to carry out the research in compliance with the General Data Protection Regulation of April 27, 2016, implemented on May 25, 2018. This research is registered in the ANSM European Union Drug Regulating Authorities Clinical Trials Database [73-2020] and at ClinicalTrials.gov [NCT04459221]. Authors obtained consent to participate in the study from participants and their parents (or holders of parental authority). Written, informed consent to participate was obtained from all participants.

### Availability of Data and Materials

The following documents relating to the research are archived by the investigator in accordance with Good Clinical Practice for a period of 15 years following the end of the research (research involving drugs, medical devices, or in vitro diagnostic medical devices or research not involving a product mentioned in article L.5311-1 of the public health code): the protocol and any amendments to the protocol, observation notebooks (copies), the source files of participants who have signed a consent form, and all other documents and letters related to the research.

Original copies of signed informed consents from participants and authority holders will be archived for a period of 30 years following the end of the research. All of these documents are the responsibility of the investigator for the regulatory archiving period. No movement or destruction will be made without the sponsor’s approval. At the end of the regulatory retention period, the sponsor will be consulted for destruction. All data, documents, and reports are subject to audit or inspection.

Within 1 year of the completion or termination of the research, a final report will be prepared and signed by the sponsor and investigator. This report will be made available to the competent authority. The sponsor will transmit the results of the research to the CPP and, if necessary, to the ANSM in the form of a summary of the final report within 1 year of the end of the research. The data sets generated and analyzed during this study are available from the corresponding author on reasonable request.

## Results

**Figure 3:**
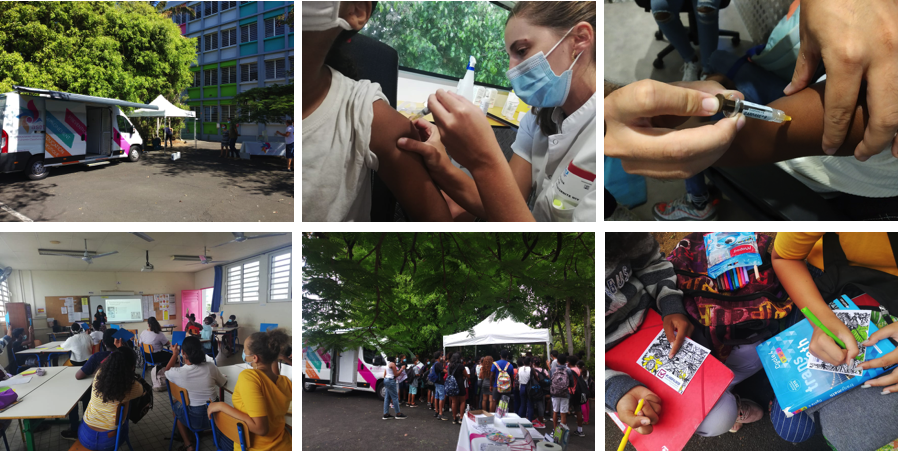
Intervention screenshots.

This study was funded in September 2019. Recruitment began in October 2020 (Figure 3). Concerning vaccination, recruitment was completed by June 2021. Concerning evaluation of satisfaction of participants and evaluation of barriers to HPV vaccination, completion of recruitment was completed by December 2021.

In the intervention school, of 780 students, 245 were randomly selected in the 12 classes. In the control school, 259 students out of 834 were randomly selected. Analyses are still ongoing, though it seems that this health promotion program offering information to students, parents, and general practitioners and free school-based vaccination had a positive impact on the intervention school and drew many students into the health bus for HPV vaccination.

## Discussion

### Principal Findings

In this study, we expect significantly higher HPV vaccination coverage (full vaccination or first dose) in the intervention school as compared to the control school, whether it be among girls or boys.

### Comparison With Prior Work

Previous studies have already shown the benefits of school-based educational sessions to improve adolescent knowledge and behavior regarding HPV prevention and increase the likelihood of the students to become vaccinated [20,21]. Education interventions represent a simple yet potentially effective strategy for increasing HPV vaccination, especially when targeting groups influential to the HPV vaccination behaviors of adolescents: parents [22], school staff [23], and health care professionals [24]. Indeed, knowledge was associated with recommendation intention and behavior.

### Strengths and Limitations

This protocol is submitted more than a year after recruitment began, since sanitary COVID-19 condition was in constant change and evolution and it was difficult to know whether we could continue the process of the trial. Amendments were made and submitted to the ethics committee, facing prohibition of meetings with more than 6 people. This protocol is the result of our constant adaptation to these different obstacles.

Having different exclusion criteria for participants in the intervention and control arms m ay introduce a selection bias by design. However, we wanted to include as many children as possible in the control group to have a representative sample of the population, and the groups may still be comparable. The sample size calculation has not taken into account correlation between participants in the same cluster. As such, the sample size is likely to be too small. However, one limit is the price of the vaccine, which limited our ability to include more students, with regard to the funds allocated.

On Reunion Island, specificities regarding economic and societal development are as follows: high rate of universal health insurance coverage where the high cost of HPV vaccine may be a barrier, mixed culture with religious faith incompatible with premarital sex and racist biopolitical mistrust of the West from which the vaccine comes from, and the particular weight of the antivaccine leagues which casted a negative halo around the subject [25]. Thus we expect a strong veto from parents.

### Future Directions

Analysis of satisfaction and specific barriers to vaccination in this school-based design will help us improve our program. Maybe on Reunion Island, with a population with early sexual life and a high rate of adolescent pregnancy (5%) [26], the target age of HPV vaccination should be reconsidered. The final implication would be an extension of this program in all middle schools on the island and an increase in HPV vaccination coverage. These results are promising and may be a stepping stone to expand this program to the whole Reunion Island and hopefully someday decrease the burden of cervical cancer.

## Appendix

Multimedia Appendix 1 Peer review report.

## Abbreviations

ANSM: Agence nationale de la sécurité du médicament
ASETIS: Association d’Education Thérapeutique et d’Intervention Sociale
CPP: Comité de Protection des personnes
HPV: human papillomavirus
SEGPA: Sections d’enseignement general et professionnel adapté
