# Supplementary material for: Impact of a Papillomavirus Vaccination Promotion Program in Middle School: Study Protocol for a Cluster Controlled Trial
Source: JMIR Res Protoc. 2022 Jun 13;11(6):e35695. doi: 10.2196/35695 (PMC9237775; doi:10.2196/35695)
Supplement: Multimedia Appendix 1 [file resprot_v11i6e35695_app1.pdf]

TRLS-D-21-01083
PROM SSCOL – Impact of a Papillomavirus vaccination promotion program in middle school: a study protocol for a randomized controlled trial
Phuong Lien TRAN; Emmanuel Chirpaz; Malik Boukerrou; Antoine Bertolotti
Trials

Dear Dr TRAN,

Thank you for considering Trials.
Peer review of your manuscript is now complete and, in the light of the reports, and my own assessment as Editor, I regret to inform you that your manuscript cannot be accepted for publication in Trials.

Please find the reviewers' reports at the end of this email. Please also take a moment to check our website at https://www.editorialmanager.com/trls/ for any additional comments that were saved as attachments.

I wish you every success with your research and hope that you will consider us again in the future.

Best wishes,

Patrick Harris, PhD
Trials
https://trialsjournal.biomedcentral.com/

Reviewer reports:

Reviewer #1: This review is to assess compliance with the SPIRIT checklist.

General comments
It is inappropriate to have different exclusion criteria for participants in the intervention and control arms, as this ensures the treatment arms are not comparable (i.e. it is introducing a selection bias by design).
The sample size calculation has not taken into account correlation between participants in the same cluster, which is necessary for cluster randomised trials. As such, the sample size is likely to be too small.
This is a cluster randomised trial, where schools act as the cluster. However, there will only be one school (cluster) per treatment arm; this is akin to having a single participant in each treatment arm. Broadly, it means we cannot perform statistical tests or calculate a measure of variance (which is required for confidence intervals or p-values) with only one cluster per treatment arm, meaning we cannot obtain any meaningful comparison between the treatment groups, apart from descriptively.

SPIRIT comments
Item 1: please clarify this is a cluster randomised trial in the title
Item 5a: please add in roles of protocol contributors
Item 5c: please add in any role the funder had in study design etc
Item 10: I think it is inappropriate to have different exclusion criteria for participants in the intervention and control arms, as this ensures the treatment arms are not comparable.

--
Please also take a moment to check our website at https://www.editorialmanager.com/trls/l.asp?i=331311&l=1VRUJKU7 for any additional comments that were saved as attachments. Please note that as Trials has a policy of open peer review, you will be able to see the names of the reviewers.

If improvements to the English language within your manuscript have been requested we recommend that you address this before submitting to another journal. We recommend that you either get your

manuscript reviewed by someone who is fluent in English or, if you would like professional help, you can use any reputable English language editing service. We can recommend our affiliates Nature Research Editing Service (http://bit.ly/NRES-HS) and American Journal Experts (http://bit.ly/AJE-HS) for help with English usage. Please note that use of an editing service is neither a requirement nor a guarantee of publication. Free assistance is available from our English language tutorial (https://www.springer.com/gb/authors-editors/authorandreviewertutorials/writinginenglish) and our Writing resources (http://www.biomedcentral.com/getpublished/writing-resources). These cover common mistakes that occur when writing in English.

This letter contains confidential information, is for your own use, and should not be forwarded to third parties.

Recipients of this email are registered users within the Editorial Manager database for this journal. We will keep your information on file to use in the process of submitting, evaluating and publishing a manuscript. For more information on how we use your personal details please see our privacy policy at https://www.springernature.com/production-privacy-policy. If you no longer wish to receive messages from this journal or you have questions regarding database management, please contact the Publication Office at the link below.

____

In compliance with data protection regulations, you may request that we remove your personal registration details at any time. (Use the following URL: https://www.editorialmanager.com/trls/login.asp?a=r). Please contact the publication office if you have any questions.
